# The feasibility, acceptability, cost and benefits of a “communities of practice” model for improving the quality of childcare centres: a mixed-methods study in the informal settlements in Nairobi

**DOI:** 10.3389/fpubh.2023.1194978

**Published:** 2023-08-01

**Authors:** Margaret Nampijja, Nelson Langat, Linda Oloo, Patrick Amboka, Kenneth Okelo, Ruth Muendo, Sabrina Habib, Martin Kiyeng, Anna Ray, Mary Abboah-Offei, Patricia Kitsao-Wekulo, Elizabeth Kimani-Murage, Jinshuo Li, Helen Elsey

**Affiliations:** ^1^African Population and Health Research Center, Nairobi, Kenya; ^2^Kidogo Innovations, Nairobi, Kenya; ^3^Department of Health Sciences, University of York, Heslington, United Kingdom; ^4^School of Health and Social Care, Edinburgh Napier University, Edinburgh, United Kingdom

**Keywords:** communities of practice, childcare centre, feasibility, benefits, quality

## Abstract

**Background:**

Informal childcare centres have mushroomed in the informal settlements of Nairobi, Kenya to meet the increasing demand. However, centre providers are untrained and the facilities are below standard putting children at risk of poor health and development. We aimed to co-design and test the feasibility, acceptability, cost and potential benefits of a communities of practice (CoP) model where trained community health volunteers (CHVs) provide group training sessions to build skills and improve practices in informal childcare centres.

**Methods:**

A CoP model was co-designed with sub-county health teams, centre providers and parents with inputs from Kidogo, government nutritionists and ECD experts and implemented in 68 childcare centres by trained CHVs. Its feasibility and potential benefits were measured quantitatively and qualitatively. Centre provider (*n* = 68) and CHV (*n* = 20) knowledge and practice scores before and after the intervention were assessed and compared. Intervention benefits were examined using linear regressions adjusting for potential confounding factors. We conducted in-depth interviews with 10 parents, 10 CHVs, 10 centre providers and 20 local government officials, and two focus groups with CHVs and centre providers. Qualitative data were analysed, focusing on feasibility, acceptability, potential benefits, challenges and ideas for improvement. Cost for delivering and accessing the intervention were examined.

**Results:**

The intervention was acceptable and feasible to deliver within existing government community health systems; 16 CHVs successfully facilitated CoP sessions to 58 centre providers grouped into 13 groups each with 5–6 centre providers, each group receiving four sessions representing the four modules. There were significant improvements in provider knowledge and practice (effect size = 0.40; *p* < 0.05) and quality of centre environment (effect size = 0.56; *p* < 0.01) following the intervention. CHVs’ scores showed no significant changes due to pre-existing high knowledge levels. Qualitative interviews also reported improvements in knowledge and practices and the desire among the different participants for the support to be continued. The total explicit costs were USD 22,598 and the total opportunity costs were USD 3,632 (IQR; USD 3,570, USD 4,049).

**Conclusion:**

A simple model delivered by CHVs was feasible and has potential to improve the quality of informal childcare centres. Leveraging these teams and integration of the intervention into the health system is likely to enable scale-up and sustainability in Kenya and similar contexts.

## Background

There is clear evidence that investing in early childhood development (ECD) during the critical period between birth and 5 years of life can have lasting benefits in the life of the child (1), reduce health inequities and boost individual, social and economic development (1–5). Increasing global focus on early childhood health and development is anchored within the Sustainable Development Goals (SDGs), particularly SDG 4 which is relevant to young children’s health, safety and development (6). Further, global leadership comes from the 71st World Health Assembly where, in 2018 the Nurturing Care Framework for Early Childhood Development (7) was established to provide a broad framework for supporting the development of children from pregnancy up to age 3. Despite this, 250 million children aged less than 5 years are at risk of not achieving their full developmental potential (1), the majority (67%) of whom are from sub-Saharan Africa (SSA). Multiple adverse exposures including poverty, malnutrition, disease, exposure to injuries and unstimulating environments underlie suboptimal child development in low- and middle-income countries (LMICs) and children living in extremely impoverished settings are particularly at risk since poverty limits access to quality health care, balanced diet, quality education and a nurturing home or preschool environment (2, 8).

Despite the increased focus on ECD, limited attention has been given to the development and provision of childcare centres in LMICs. The focus on childcare is particularly important in this era of rapid urbanization, with over half the world’s population living in urban settings. It is estimated that by 2050, 56% of the population in Africa and 64% in Asia will be living in towns and cities (9). Urbanization brings with it social, economic and cultural changes and has been identified, in itself, as a determinant of health (10). Rapid urbanization has brought changing work patterns with increases in female employment outside the home, resulting in a pressing need for childcare options particularly in low-income urban settings. The changing socio-cultural context in the urban settings in Kenya and in similarly rapidly urbanising cities provide limited options for childcare. Child care for children 0–3 years (preschool age) is commonly provided by the mother (or less often other family member) at home or at her workplace, or by paid childcare in centers where children are kept during the day while their mothers are out working. In these centers, children are expected to be fed, kept healthy and clean, and to have a stimulating environment for learning. There are also informal arrangements where a neighbour is requested to take care of the child (11, 12). The choice on which strategy is used is determined by the family socio-economic factors and the broader context of living in urban informal settings (12). Unlike in the rural settings, families in the cities have no extended family around, and this means that that women who are the primary and often sole caregivers for children often opt for paid childcare, yet they live on a meagre pay which only affords cheap and low quality childcare services for their children.

Quality childcare centres have the potential to provide multiple benefits to children, families and communities (7) through enabling women’s participation in the labour force (13–17). There are bi-directional benefits as increases in women’s employment have the potential to provide indirect benefits to the child through increased household income and improved nutrition (18). A framework for providing quality childcare is found in WHO’s Nurturing Care guidelines which specifies that an environment should be healthy, safe, hygienic, provide nutritious food and responsive nurturing care (7). Ensuring that centre providers understand these elements and can apply them to their centres is critical for programmes that train and support centre-based care providers. While there are still significant gaps in the evidence of impacts of childcare on children’s cognitive, socio-emotional and physical health in high-income and particularly, LMIC contexts (18, 19), the role of a responsive caregiving and nurturing environment is increasingly recognized and emphasized (20). A well-facilitated childcare centre that provides opportunities for learning and play, good feeding and promotes good health has the potential to nurture and optimize child development (21–24). On the other hand, childcare centres with limited cognitive stimulation are likely to hold back children’s development (25).

While there are gaps in policy provision in Kenya, the Government of Kenya has outlined guidelines for childcare centres such as the Early Childhood Development Service standard guidelines that were instituted in 2006 (26). However, due to lack of resources, limited training, low supervision and absence of assessment tools, many childcare centres do not meet the minimum standards of care (27) specified in the Kenya ECD guidelines (26). This is particularly the case in informal settlements where providers, who are almost exclusively women, are frequently untrained and unsupported. Care is offered in one or two rooms with limited facilities to provide a hygienic, safe and stimulating environment. Estimates of the number of such childcare centres in informal settlements in Nairobi put the figure at 2700 (27) but should currently be much more than this. Therefore, many children are at risk of receiving inadequate care and nurturing during the critical period of their development, which in turn is detrimental for their future learning and wellbeing (28). Given the poor quality of childcare centres in LMICs particularly in impoverished settings due to lack of resources, lack of skills on the part of the childcare centre providers and absence of clear guidelines to regulate centre-based childcare, our programme of research aimed to support the improvement of the quality of centre-based childcare in line with the WHO’s nurturing care guidelines (7). The intervention was co-designed by centre providers, community health teams, parents, local and national government and is described in detail in a sister paper (29). This study aimed to assess the feasibility, acceptability, cost and benefits of the CoP intervention for improving the quality of childcare centres. Our expectation was that by imparting the childcare centre providers with the necessary knowledge and skills of childcare provision they would improve the quality of care they provide.

## Methods

### Study design

**Figure 1:**
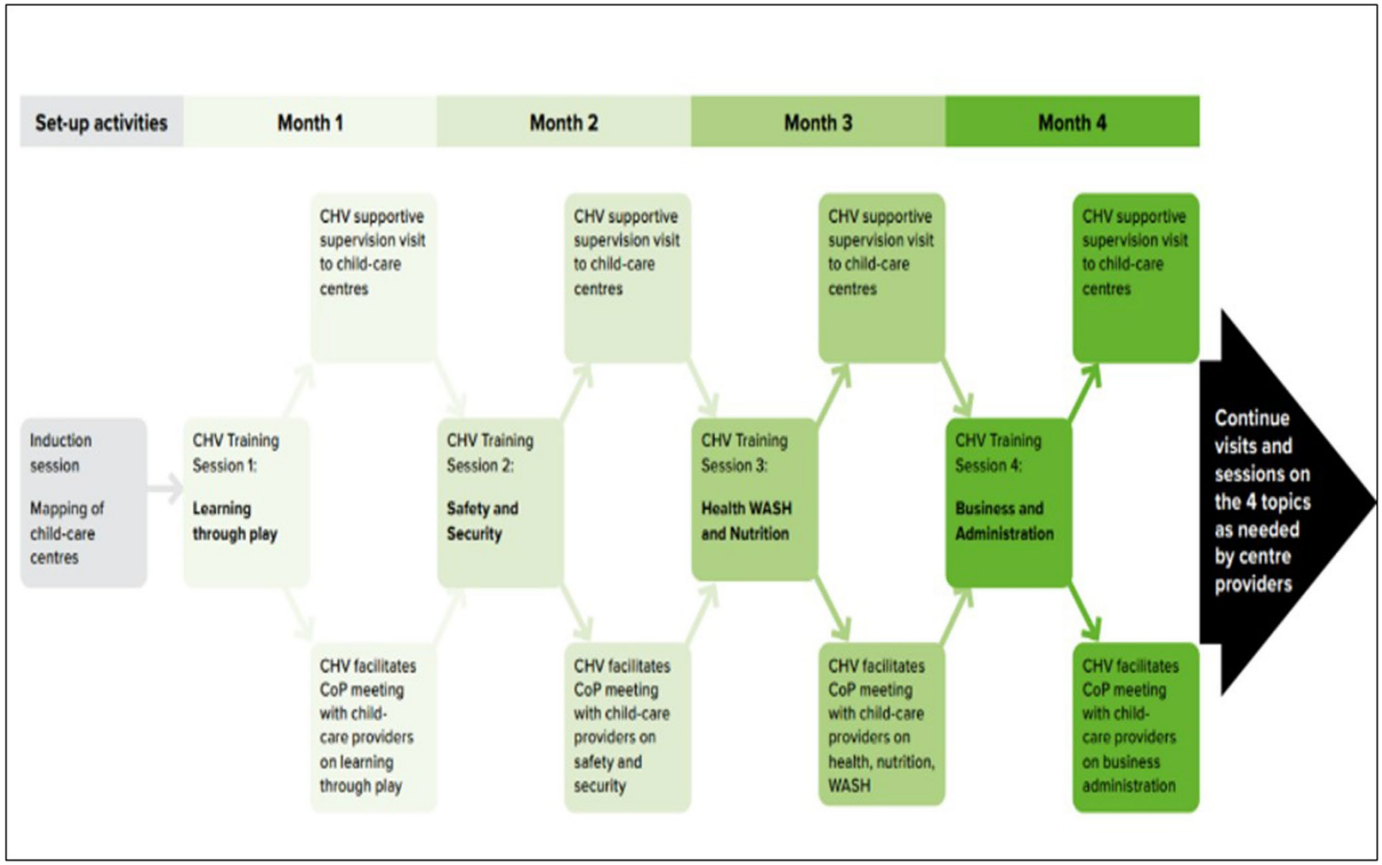
The planned intervention process. This shows the sequence of how the different data collection and co-design activities were undertaken. Activities that come earlier (on top) inform subsequent activities and so on.

This was an uncontrolled pre-post study that utilized both quantitative and qualitative methods. It was the final phase of a three phase study which employed a sequential mixed-methods (30) design (illustrated in Figure 1). The overall study aimed to answer the following objectives: (1) To map and assess the childcare environment and provider skills in informal settlements; (2) To co-design with childcare providers, parents, government and ECD experts a supportive assessment and skills-building community of practice (CoP) approach which can be delivered at scale within informal settlements in Kenya; (3) To assess the feasibility acceptability, benefits and costs of delivering the co-designed model over a six-months’ period in two informal settlements in Nairobi (Korogocho and Viwandani). Here we report the findings of objective 3 covering the final evaluation of feasibility acceptability, benefits and costs of the co-designed model. The codesign process is described in Oloo et al. (29) and the findings on the factors influencing the quality of childcare centres are presented in Nampijja et al. (awaiting publication). In this study, we aim to test the feasibility, acceptability, benefits and the cost of a co-designed CoP model on the centre quality, and the knowledge and skills of the centre providers and CHVs.

### Study setting

The study was conducted in two informal settlements (Korogocho and viwandani) in Nairobi, Kenya. These settlements were selected as their population includes a high proportion of women working outside the home the majority of whom work for a daily wage in the informal sector.

The socio-demographics profiles of these communities have been well characterized by the Nairobi Urban Health and Demographic Surveillance System (NUHDSS) (31), within the African Population and Health Research Center, (APHRC). Korogocho and Viwandani, located about 7 km from each other, are densely populated with 63,318 and 52,583 inhabitants per square km, respectively. The settlements are characterized by poor housing, poor sanitation, lack of basic infrastructure, insecurity, high crime rate and poor access to maternal and child health (MCH) services and health care in general (31). The two communities were selected because they represent the poverty spectrum on which informal settlements in Nairobi lie, with Viwandani (which is close to the industrial area) being relatively less poor than Korogocho (31, 32). This variation enables the transferability of our findings to a wide range of urban-poor settings in sub-Saharan Africa.

To meet the childcare needs of families in these settlements, there are many informal, low quality, but affordable childcare centres (Nampijja et al. awaiting publication – predictors paper). Following our initial mapping exercise, we categorised childcare centres as: (i) faith-based run by and often located within religious institutions, (ii) centre-based, small centers often run small centres often run by NGOs or private organisations, (iii) home-based run from within a resident’s (predominantly women) own house with limited facilities or training, and (iv) school-based attached to a primary school. Following the mapping of childcare centres and co-design workshops with local government, community health teams, parents and childcare centre providers, school-based centres were excluded from the intervention implementation (29). This allowed the intervention to focus on the lowest quality and least supported home-based, and small centres (faith-based and centre-based).

### The intervention and its implementation

The co-designed intervention includes training of CHVs to deliver a series of monthly group meetings with approximately six childcare providers in their catchment areas (each CHV is responsible for approximately 100 households in which they routinely support health care programmes). CHV training and subsequent CoP meetings cover: (i) learning through play including making toys from locally available material, (ii) safety and security including child safeguarding, (iii) essential child health, sanitation, hygiene, and nutrition, and (iv) business management including tracking income and expenditure (see Figure 1). Group meetings are facilitated by the CHVs and run as communities of practice (33) to allow centre providers to share experiences and apply learning to their own centre context. In between the CoP meetings, CHVs visit centres to support them to implement what they have learned. Details of the intervention are specified according the TiDieR guidelines (34) in the Supplementary materials. The implementation manual for the intervention can be found here https://www.york.ac.uk/media/healthsciences/documents/research/public-health/Implementation%20Manual.pdf.

Following the initial mapping (29) we conducted baseline assessments of 68 centres (16 Korogocho and 52 Viwandani). All were invited to participate in a six-month pilot of the intervention and all accepted. While we planned 6 months for the pilot, due to COVID 19 restrictions, the pilot had to be completed within 5 months. Following discussion with the CHVs and providers, 13 groups of CHVs each comprising 5–6 centre providers were established, hence we had 3 groups from Korogocho and 10 from Viwandani. Further changes to the planned intervention were required due to COVID restrictions and the shortened time-frame for implementation. The supervisory visits to the centres by the CHVs which were initially planned to be done every after a module, only began in month 3 of the intervention and only two visits to each centre were possible. During the visit, CHVs monitored any changes in the childcare environment including care providers’ skills and practices, and advised childcare providers as necessary. A short and simple assessment tool developed during the co-design phase, was used to collect these data. It had 22 items including three on child safety and stimulation; three on responsive caregiving; three on learning through play; two on health; two on nutrition; four on WASH; two on parental engagement; and two on management and administration. Advice was given to the centre provider based on which areas needed more support. Certificates were given to centre providers and CHVs involved in the intervention as a means of recognising their involvement and to provide motivation. More information on the intervention are available in a related manuscript (29).

### Outcomes definition and measurement

Study outcomes included those related to the implementation process, i.e., the feasibility and acceptability of the intervention; potential benefits of the intervention, and cost of its implementation. Feasibility and acceptability were assessed using data from the simple assessment tool, and implementation observations as well as qualitative interviews that captured perspectives of community health teams (local government, CHAs and CHVs), centre providers and parents. Intervention benefits were measured quantitatively using centre provider and CHV knowledge and practices scores, and centre quality scores. A costing tool was used to capture all relevant implementation costs. The outcome measures are summarised in Table 1, and the detailed procedures described in the section that follows.

**Table 1:** Outcomes measures and tools used.

Outcome	Tools	Time points
Feasibility and acceptability	Simple assessment tool	Two supervisory visits during the intervention
	Implementation data (observations)	Periodically during intervention implementation
	Qualitative interviews with Centre providers, CHVs, CHAs and County officials	Baseline and endline
Benefits of the intervention	CHV KAP tool	Baseline and endline
	Centre provider KAP tool	Baseline and endline
	Centre quality assessment tool	Baseline and endline
	Qualitative interviews with Centre providers, CHVs, CHAs and County officials	Baseline and endline
Cost	Costing tool	During implementation period

### Assessing feasibility, acceptability, and potential benefits of the intervention

#### Quantitative procedures

*Feasibility and acceptability assessments*: We analysed the results of the visit assessments conducted by CHVs and documented all aspects of the intervention including (1) the training and supervision of CHVs who deliver the intervention; (2) the number of supportive supervision visits made by each CHV to childcare centres; (3) the number of CoP groups established and sessions run, a record of participants in each group (number and names), the duration and topics covered.

To gain a more detailed assessment of the feasibility and acceptability, and content of the supportive supervision and CoP sessions, the research team observed eight CoP sessions (i.e., two for each of the four CoP groups) and 10 childcare centre visits over the 5-month implementation period. These observations followed an observation guide to identify facilitators and barriers facing the childcare providers in implementing the recommended skills and practices and reflections on the interaction between the CHVs and childcare providers. These were assessed by a team of trained field interviewers using Kidogo tools that were adapted to the informal settlements.

*Assessing intervention benefits:* Baseline and endline assessments were, respectively, conducted in February and October 2021. The assessments included: (1) Knowledge and practices questionnaire for CHVs and providers on the key domains child protection, responsive caregiving, learning through play, health, nutrition, WASH, and business administration. (2) Detailed quality assessment of the centre environment based on the same domains assessed in the CHV and centre provider KAP. (3) Implementation data: We recorded of numbers of participants that attended each session, the number of sessions delivered, visits made to by each CHV. This information was collected by our research team and CHVs. KAP assessments were conducted virtually, while centre environment and observations of the sessions were done face-to-face.

#### Qualitative procedures

In line with our sequential mixed methods approach, the results of the questionnaires and changes in knowledge, attitude and practice scores were used to sample 10 childcare providers for qualitative interviews. The aim was to understand experiences from those who had improvement in their knowledge and practices, and those who did not. The interviews focused on the acceptability of the intervention and any challenges and barriers providers faced in improving quality in their childcare centres. Ten CHVs were selected based on their questionnaire scores to participate in interviews and also in two focus groups following 5 months of implementation. The interviews with CHVs captured their personal experiences of the training and implementation of the intervention. The focus groups allowed a more detailed discussion on the barriers and facilitators to implementation, possible improvements and potential sustainability within the community health system.

Because of the COVID-19 pandemic and related restriction on face-to-face interactions, in-depth interviews and KIIs were conducted virtually, while the FGDs were done face-to-face. The blended approach with face-to-face and remote options of data collection and engagements minimized physical contact and hence risk to infection transmission so that over the entire duration of the study, there was no case of COVID -19 reported among our teams and participants. We took extra measures to ensure that quality data was collected especially in the case where telephone or zoom interactions were conducted. These measures included spot checks with participants, and reviewing the data to ensure completeness of the questionnaires.

We conducted in-depth interviews with 10 parents whose children used the childcare centres involved in the CoP sessions to understand if they had noticed any changes or had any feedback on the intervention. We interviewed 20 local government officials at different levels including national level, county level and sub-county level to identify any facilitators and barriers to implementation and their views on integration within the work of community health teams and possibilities for scale-up.

The number of respondents used for the different qualitative interviews were considered to be sufficient to provide representative views. For the policy makers and implementers interviews, all the participants who were involved in the intervention development were interviewed. However, for the CHVs and centre providers and parents, we selected 10 participants from each group with a representation from the two locations, and inclusion of CHVs, centre providers and parents from centres which had high, moderate and low scores in quality and skills on childcare at the end line assessments. Sample size of 10–20 participants included in the qualitative interviews for each group were considered sufficient to provide the required information. Purposeful sampling based on specific criteria allowed collection of balanced information on the participants’ experiences of the intervention.

Qualitative in-depth interviews and focus groups were conducted by two researchers (LO and PA) experienced in qualitative methods and data collection with a detailed knowledge of the intervention and the context of childcare within the informal settlements. Interviews with providers were conducted in their centres in Swahili; interviews with parents were conducted in their homes in Swahili, and CHVs were interviewed at a community venue in Swahili while KII with Local government officials were interviewed in English both face-to-face (in their offices) and on phone/virtual. The focus groups were held in community centres. All interviews and focus group discussions were audio-recorded and transcribed as soon as possible after they were held. Interviews and focus group discussions were conducted in Swahili and later transcribed and translated for analysis.

### Assessing costs of the intervention

We documented (1) number and duration of the training and supervision of CHVs and CHAs who delivered the intervention; and the number of trainers and trainees attended; (2) the number of supportive supervision visits made by each CHV to childcare centres and their duration; and (3) the number of CoP groups established and sessions run, a record of CHVs, CHAs and participants in each group (number and names), the duration and topics covered.

The explicit costs included fees, expenses or allowances paid for the training for the intervention (venue, refreshments, printing, trainers, CHAs and CHVs), and, delivery of the CoP sessions and follow-up supervisory visits (CHVs and care providers if applicable), including those borne by other partners. These amounts were recorded by the study team as part of financial records. We also estimated opportunity costs of time of the personnel involved by multiplying their respective hourly wage by their working time on the intervention. They were collected in local currency: Kenyan Shillings (KSh) 2021 prices and presented alongside USD for total costs (1 USD = 109.64 KSh) [IMF (2022). Exchange rates selected indicators (Internet). Available at: https://data.imf.org (Accessed March 7, 2022)].

### Data management and analysis

#### Calculation of outcome scores

*Quality of childcare centres scores:* The quality of childcare centres was measured using a set of questionnaire items. The tool focused on nurturing care framework components namely: (i) child protection, safety, discipline and abuse, (ii) stimulating environment, (iii) responsive caregiving, (iv) learning through play, (v) health, (vi) nutrition, (vii) water, sanitation, and hygiene. Additionally, the items included the (viii) business and administration component which focused on the capability of centre providers to provide quality service while earning an income. Varying number of items was asked under each component. One score was assigned for each positive/correct response to an item and a score of zero otherwise. Then the total score was calculated for each component by adding up the scores corresponding to each item in that component. The component scores were converted to percentages to make them more intuitive. To obtain a component score of a childcare centre, their total score in that component was divided by the maximum possible score of that component and multiplied by 100. The overall quality score of a childcare centre was the mean of the individual component scores, that is, the sum of all component scores divided by the number of components. Details of the childcare centre quality tool and scoring system are provided in Appendix 2.

*Childcare providers KAPs scores:* The childcare providers who agreed to the quality assessment visits were administered questionnaires to assess their knowledge and skills on nurturing care and business. The components of the questionnaire were similar to the quality assessment tool and included the following: (i) child protection, safety, discipline and abuse, (ii) stimulating environment, (iii) responsive caregiving, (iv) learning through play, (v) health, (vi) nutrition, (vii) water, sanitation, and hygiene and (viii) business and administration. The response to each question/item was assigned a score of one if it was positive/correct and zero otherwise (see Appendix 3). Component scores and overall childcare provider KAPs score were obtained the same way as the quality of childcare centre scores. Details of the childcare provider KAPs tool and scoring system are provided in Appendix 3.

*CHVs KAPs scores:* The CHVS were assessed for their knowledge and skills around the nurturing care framework and their perceived competence on providing support supervision. Apart from the nurturing care components, the CHV KAPs questionnaire included two more components: providing support supervision of centre providers, and attitude and perceived competence to provide support. Each questionnaire item was then assigned a score of one if positive/correct and zero otherwise (Appendix 4). The component scores and the overall CHV KAPs score were obtained in a similar way as the quality of care and care provider scores. Details of the CHV KAPs tool and scoring system are provided in Appendix 4.

#### Data analysis

Quantitative cost and qualitative interviews focused on the feasibility, acceptability cost and benefits of the intervention. Accordingly, a mixed methods approach was used to analyse the different outcomes. Feasibility was mainly measured through qualitative data, however, quantitative indicators including numbers of participants who completed the program and number of sessions done were analysed.

*Quantitative data analysis:* Data management and analysis was done using STATA version 17. Descriptive statistics were used to summarize the data from the quality assessment tool and the knowledge and skills of centre providers and CHVs. Continuous variables were summarized using means (SD) and medians (IQR) depending on their distribution. Categorical variables were summarized using frequencies and percentages. These descriptive statistics were presented in tables. Bivariate analysis was done to compare outcomes between baseline and endline, e.g., comparison of centre quality scores before and after the intervention. To compare continuous variables, e.g., provider KAP score between baseline and endline, paired sample t-test was used since the study was uncontrolled pre-post. Comparison of proportions of binary variables (e.g., proportion of centres with a handwashing station) between surveys was done using paired proportions t-test. To evaluate the potential benefits of the co-designed CoP model on the centre quality, and the knowledge and skills of the centre care providers and CHVs, linear mixed effects regression model was used. The random effects variable in the models was the unique participant IDs. This model is appropriate for this analysis because in the determination of the association between the outcome and the exposure, it accounts for the correlation of repeated measures on of an individual. Simple linear mixed effects regression was used to obtain crude effects while adjusted effects were obtained from the multiple linear mixed effects regression model. For each of the three outcome variables namely: centre provider KAP score, centre quality score, and CHV KAP score, separate models (both crude and adjusted) were fit. The main independent variable in each of the three models was the survey round (pre-, post-intervention). In both centre provider KAP and centre quality models, the adjusted analysis controlled for the type of centre (home-based, centre-based, faith-based), centre provider age (years), centre provider highest education (primary, secondary, tertiary), location of the childcare centre (Korogocho, Viwandani), period of operation (years), and centre provider ECD training (yes, no). In the CHV KAP model, the adjusted analysis controlled for CHV age (years), CHV sex (female, male), CHV highest education level (primary, secondary, tertiary), and CHV area of operation (Korogocho, Viwandani). The effect size (standardized coefficient) and the corresponding *p*-values and the 95% confidence intervals were reported. Before running the regression models, each of the outcome scores were standardized by subtracting the baseline mean score from each observed score and dividing this by the standard deviation of the baseline score, e.g., to standardize the score of a centre provider KAP score, the baseline (the “control arm” in this study) centre provider KAP mean score was subtracted from her score and the result divided by the standard deviation of the centre provider KAP score. The coefficients from the regression were interpreted in terms of standard deviation differences (pre- vs. post-intervention) rather than mean differences.

*Qualitative data analysis:* An initial round of analysis of all qualitative data was conducted by six members of the team (LO, HE, PA, AR, PK-W, MN, and KO) to develop a coding frame. Following discussions among the team, the analysis framework was agreed and applied to all qualitative data by LO and PA. The framework included themes on feasibility, acceptability and experiences of the intervention as well as reflections on the potential for scaling up the intervention. Nvivo 2020 was used to organise the qualitative analysis. We compared, collated/triangulated the information from these different data sources, both quantitative and qualitative to understand acceptability of the intervention (35). The extent of convergence, divergence or silence between findings was identified following the development of an integrated results matrix (see Table 2) (36).

**Table 2:** Characteristics of participants who participated in qualitative interviews.

	Attribute name										
	Gender		Location		Seniority		Sector		Level		
	Male	Female	Viwandani	Korogocho	Junior official	Senior official	Health	Education	National	County	Sub county
Centre providers	0	10	7	3							
CHVs	2	8	7	3							
Parents	0	10	7	3							
Policy makers	8	12			11	9	15	5	4	4	12

The meta-inferences were devised and discussed across our full team and shared with the stakeholders involved in the supportive assessment and CoP model throughout the study in a final dissemination workshop to ensure that our interpretation of the data adequately reflects their perspectives. The evaluation and the final inputs from stakeholders informed a final version of an implementation manual to facilitate the intervention to be integrated within the existing CHV structure and scale up of the model to other parts of Nairobi and countrywide.

## Results

### Profile of the childcare centres

A total of 58 centres completed the sessions and took part in the endline survey across the two informal settlements with 13 (22%) in Korogocho, and 45 (78%) in Viwandani (Table 3). Of these, 40 (69%) were home based and 11 (19%) centre based (autonomous centres operating in buildings purposely built for provision of childcare services) and 7 (12%) were faith-based centres. Home-based centres tended to have younger children aged 0–3 years (62%), while other types of centres had more children older than 3 years. Centre provider to child ratio was smaller among home based centres with a mean of 1 centre provider to 7 children, but in the other centres it ranged between 15 and 21 children for one centre provider. The ratio of boys to girls in the centres was fairly balanced and similar across the centres. Whereas most of home-based centres had been in operation for 0–2 years, a majority of centre-based and faith-based centres had been in operation for more than 2 years. The median amount charged per day for each child varied between the different types of centres, ranging from Ksh. 30 in the centre-based centres to Ksh. 50 in the home-based centres. The median charges per day for all the childcares was Ksh. 50, ranging from Ksh. 10 to Ksh. 100. One-tenth of the centres (10%) reported that they were supported by an organization. Overall, less than half (38%) of the centre providers had ECD training, with faith-based (86%) and centre-based (73%) centres having the highest proportion of trained providers while home-based (20%) had the least.

**Table 3:** Profile of the childcare centres.

Variable	Category/summary statistic	Home based (*N* = 40)	Centre based (*N* = 11)	Faith based (*N* = 7)	Total (*N* = 58)
Location	Korogocho, *n* (%)	5 (13)	5 (45)	3 (43)	13 (22)
	Viwandani, *n* (%)	35 (88)	6 (55)	4 (57)	45 (78)
Number of children in the centre	Median (IQR)	7 (4–11)	26 (15–36)	33 (20–54)	10 (5–20)
	[Range]	[1–25]	[7–60]	[11–105]	[1–105]
Number of children 0–3 years old	*n* (%)	274 (62.3)	116 (26.4)	50 (11.4)	440 (47.7)
Sex (boys)	*n* (%)	166 (49.2)	137 (46.1)	134 (48.2)	437 (47.9)
Provider to child ratio	Median ratio	1:7	1:15	1:21	1:8
Years of operation	0–2 years, *n* (%)	21 (53)	4 (36)	1 (14)	26 (45)
	3–5 years, *n* (%)	4 (10)	3 (27)	3 (43)	10 (17)
	6–10 years, *n* (%)	10 (25)	2 (18)	1 (14)	13 (22)
	>10 years, *n* (%)	5 (13)	2 (18)	2 (29)	9 (16)
Charges per day (KES)	Median (IQR)	50 (50–67)	30 (20–50)	40 (15–50)	50 (40–50)
	[Range]	[30–100]	[10–100]	[10–50]	[10–100]
Received support from any organisation	*n* (%)	4 (10)	1 (9)	1 (14)	6 (10)
Provider trained in ECD	*n* (%)	8 (20)	8 (73)	6 (86)	22 (38)
Provider interested in supervision visits	*n* (%)	40 (100)	11 (100)	7 (100)	58 (100)
Provider interested in group meetings	*n* (%)	40 (100)	11 (100)	7 (100)	58 (100)

### Socio-demographic characteristics of the childcare providers

Of the 129 centres identified, school based centres were excluded as they were not identified as a priority during the codesign process. The total number of home-based, small centre-based and faith-based centres eligible for detailed assessment was 68. Out of the 68 eligible childcare centres, 66 were surveyed at baseline while the other two were not reached. The 66 were included in the CoP sessions in Korogocho and Viwandani between March 25 and April 13, 2021, out of which 58 had complete data in both baseline and endline surveys. These 58 centres/providers formed our panel for analysis. There were no significant differences in the baseline socio-demographic characteristics and outcome variables between the 58 care providers in the panel and the 8 who were not reached at endline (Appendix 1). All the 17 CHVs who took part in the intervention were interviewed in both surveys.

About three-quarters (74%) of the childcare centres were in Viwandani and almost all (98%) the centre care providers were female (Table 4). The mean age of the centre providers was 40 years, ranging from 23 to 63 years. Most of the care providers (43%) had primary education.

**Table 4:** Centre provider socio-demographic characteristics.

	Home based (*N* = 40) *n* (%)	Centre based (*N* = 11) *n* (%)	Faith-based (*N* = 7) *n* (%)	Total (*N* = 58) *n* (%)
Location of centre				
Korogocho	7 (18)	5 (45)	3 (43)	15 (26)
Viwandani	33 (83)	6 (55)	4 (57)	43 (74)
Age (years)				
Mean (SD)	39.1 (7.7)	38.4 (8.1)	38.3 (12.6)	38.8 (8.3)
[Range]	[22–53]	[27–54]	[27–59]	[22–59]
Sex				
Female	40 (100)	10 (91)	7 (100)	57 (98)
Male	0 (0)	1 (9)	0 (0)	1 (2)
Highest education level				
None	0 (0)	0 (0)	0 (0)	0 (0)
Primary	24 (60)	2 (18)	1 (14)	27 (47)
Secondary	14 (35)	3 (27)	3 (43)	20 (34)
Tertiary	2 (5)	6 (55)	3 (43)	11 (19)

### Childcare centre quality scores

Overall, there was a significant improvement in the centre quality score from 59% [95% CI: (56, 62)] at baseline to 66% [95% CI: (63, 69)] at endline (*p* = 0.002) (Table 5). There was a significant positive difference in three out of four domains measuring centre environment quality: Child protection, child safety, child abuse and positive discipline stimulating environment domain (from 67% to 78%; *p* = 0.001), learning through play domain (from 25% to 37%; *p* = 0.019), and business administration domain (from 33% to 63%; *p* < 0.001). Considering the type of centre, there was a significant and positive difference in the quality of care score in the home-based centres (from 55% to 65%; *p* < 0.001) while in the other two types, the differences were not significant (see Table 5).

**Table 5:** Baseline and endline centre quality scores.

	Home based (*N* = 40)			Centre based (*N* = 11)			Faith based (*N* = 7)			Total (*N* = 58)		
	Baseline	Endline	*p*-value	Baseline	Endline	*p*-value	Baseline	Endline	*p*-value	Baseline	Endline	*p*-value
Responsive caregiving												
≤15 children per provider; *n* (%)	40 (100)	40 (100)	NA	9 (82)	9 (82)	1.000	6 (86)	4 (57)	0.237	55 (95)	53 (91)	0.464
Daily routine is planned, posted and used; *n* (%)	1 (3)	5 (13)	0.090	4 (36)	3 (27)	0.647	3 (43)	3 (43)	1.000	8 (14)	11 (19)	0.452
Responsive care giving subtotal score; mean (SD)	100.0 (0.0)	100.0 (0.0)	NA	90.9 (20.2)	86.4 (32.3)	0.676	85.7 (37.8)	78.6 (26.7)	0.604	96.6 (15.8)	94.8 (18.0)	0.484
Play and early learning												
Children have something to play with; *n* (%)	6 (15)	24 (60)	**<0.001**	4 (36)	4 (36)	1.000	7 (100)	7 (100)	NA	10 (17)	28 (48)	**<0.001**
Separate area with play materials, toys, books, pens; *n* (%)	9 (23)	16 (40)	0.091	10 (91)	8 (73)	0.269	6 (86)	2 (29)	**0.031**	25 (43)	26 (45)	0.852
Early learning subtotal score; mean (SD)	13.3 (22.4)	37.5 (32.2)	**<0.001**	54.5 (27.0)	45.5 (27.0)	0.277	42.9 (25.2)	23.8 (31.7)	0.231	24.7 (29.0)	37.4 (31.3)	**0.019**
Child protection												
Child protection, child safety, child abuse and positive discipline stimulating environment; mean (SD)	63.6 (18.8)	75.4 (19.1)	**0.004**	74.0 (10.7)	84.4 (21.6)	0.136	75.5 (24.3)	79.6 (16.2)	0.703	67.0 (18.8)	77.6 (19.3)	**0.001**
Health												
Centre has a first aid kit; *n* (%)	1 (3)	5 (13)	0.089	4 (36)	3 (27)	0.647	0 (0)	1 (14)	0.299	5 (9)	9 (16)	0.254
Thermometer and records of temperature check available; *n* (%)	1 (3)	1 (3)	1.000	3 (27)	1 (9)	0.269	3 (43)	1 (14)	0.237	7 (12)	3 (5)	0.186
Checks child health daily and knows what to do if sick; *n* (%)	35 (88)	36 (90)	0.723	10 (91)	10 (91)	1.000	7 (100)	5 (71)	0.127	52 (90)	51 (88)	0.768
Knows immunisation status of children; *n* (%)	26 (65)	34 (85)	**0.039**	8 (73)	10 (91)	0.269	2 (29)	3 (43)	0.577	36 (62)	47 (81)	**0.024**
Health subtotal score; mean (SD)	45.7 (16.9)	48.6 (17.7)	0.479	62.3 (25.8)	53.2 (20.3)	0.295	59.2 (12.9)	38.8 (13.6)	0.058	50.5 (19.5)	48.3 (17.9)	0.524
Water, sanitation and hygiene												
Handwashing station: water and soap; *n* (%)	26 (65)	23 (57)	0.491	11 (100)	10 (91)	0.306	7 (100)	6 (86)	0.299	44 (76)	39 (67)	0.303
At least one potty for every 5 children; *n* (%)	35 (88)	33 (83)	0.531	8 (73)	8 (73)	1.000	4 (57)	4 (57)	1.000	47 (81)	45 (78)	0.647
Centre is cleaned daily and visibly clean; *n* (%)	39 (98)	39 (98)	1.000	10 (91)	11 (100)	0.306	6 (86)	4 (57)	0.237	55 (95)	54 (93)	0.697
WASH subtotal score; mean (SD)	83.8 (19.2)	79.4 (18.7)	0.343	86.4 (20.5)	86.4 (17.2)	1.000	82.1 (18.9)	57.1 (27.8)	0.062	84.1 (19.1)	78.0 (21.0)	0.114
Nutrition												
Children receive morning uji (porridge); *n* (%)	37 (93)	34 (85)	0.289	10 (91)	10 (91)	1.00	7 (100)	7 (100)	NA	54 (93)	51 (88)	0.342
Receive lunch; *n* (%)	38 (95)	37 (93)	0.644	10 (91)	11 (100)	0.306	7 (100)	7 (100)	NA	55 (95)	55 (95)	1.000
Children are served with warm food; *n* (%)	35 (88)	40 (100)	**0.021**	9 (82)	11 (100)	0.138	6 (86)	6 (86)	1.00	50 (86)	57 (98)	**0.015**
Poster of a balanced diet is displayed; *n* (%)	1 (3)	3 (8)	0.305	0 (0)	2 (18)	0.138	0 (0)	0 (0)	NA	1 (2)	5 (9)	0.094
Nutrition subtotal score; mean (SD)	69.4 (17.4)	71.3 (15.6)	0.596	65.9 (23.1)	77.3 (13.5)	0.176	71.4 (9.4)	71.4 (9.4)	1.000	69.0 (17.7)	72.4 (14.6)	0.231
Business administration												
Attendance register kept and available; *n* (%)	16 (40)	33 (83)	**<0.001**	10 (91)	10 (91)	1.000	7 (100)	5 (71)	0.127	33 (57)	48 (83)	**0.002**
Track finances with records; *n* (%)	12 (30)	32 (80)	**<0.001**	9 (82)	11 (100)	0.138	6 (86)	7 (100)	0.299	27 (47)	50 (86)	**<0.001**
Centre; *n* (%) policies/fees/schedules clearly posted; *n* (%)	0 (0)	7 (18)	**0.006**	1 (9)	3 (27)	0.269	1 (14)	2 (29)	0.515	2 (3)	12 (21)	**0.004**
Budget available; *n* (%)	7 (18)	22 (55)	**0.001**	4 (36)	8 (73)	0.087	4 (57)	6 (86)	0.237	15 (26)	36 (62)	**<0.001**
Business administration subtotal score; mean (SD)	21.9 (27.3)	58.8 (29.7)	**<0.001**	54.5 (21.8)	72.7 (17.5)	0.070	64.3 (24.4)	71.4 (22.5)	0.631	33.2 (30.8)	62.9 (27.4)	**<0.001**
Overall centre environment quality score (percent of correct responses)												
Mean (SD) centre quality score (%)	55 (10)	65 (11)	**<0.001**	69 (11)	72 (12)	0.391	68 (2)	60 (11)	0.093	59 (12)	66 (11)	**0.002**

The bold figures are *p*-values less that 0.05.

### Centre provider knowledge and practice scores

There was a significant positive improvement in the overall centre provider KAP score from 72% to 77% (*p* = 0.005). This improvement was driven mainly by the business management (from 56% to 75%; *p* < 0.001) and child nutrition (from 69% to 80%; *p* = 0.001) domains, each recording significant positive differences. Among the three centre types, only home-based centres registered a significant (positive) difference in the mean centre provider KAP scores (from 69% to 76%; *p* = 0.001) (Table 6).

**Table 6:** Baseline and endline centre provider KAP scores.

Centre provider knowledge and practice scores (percent of correct responses)												
Centre type	Home based (*N* = 40) Mean (SD)			Centre based (*N* = 11) Mean (SD)			Faith based (*N* = 7) Mean (SD)			Total (*N* = 58) Mean (SD)		
Time point	Baseline	Endline	*p*-value	Baseline	Endline	*p*-value	Baseline	Endline	*p*-value	Baseline	Endline	*p*-value
Business management	48.0 (21.7)	72.3 (16.9)	**<0.001**	72.7 (13.5)	84.5 (6.9)	**0.024**	78.6 (6.9)	78.6 (22.7)	1.000	56.4 (22.8)	75.3 (16.8)	**<0.001**
Child safety	100.0 (0.0)	100.0 (0.0)	NA	100.0 (0.0)	100.0 (0.0)	NA	100.0 (0.0)	100.0 (0.0)	NA	100.0 (0.0)	100.0 (0.0)	NA
Responsive caregiving	55.8 (38.8)	73.3 (33.9)	**0.026**	75.8 (39.7)	84.8 (27.3)	0.574	66.7 (27.2)	52.4 (46.6)	0.589	60.9 (38.1)	73.0 (35.0)	0.077
Learning through play	56.6 (17.5)	58.0 (16.4)	0.726	76.0 (9.3)	74.4 (14.6)	0.617	74.0 (12.2)	68.8 (11.6)	0.493	62.4 (17.8)	62.4 (16.8)	1.000
Child health	72.2 (18.1)	69.4 (16.5)	0.372	77.8 (19.2)	72.7 (17.5)	0.518	63.5 (16.6)	52.4 (17.8)	0.062	72.2 (18.3)	68.0 (17.5)	0.111
Child nutrition	67.5 (19.6)	78.0 (14.2)	**0.002**	76.4 (19.6)	87.3 (16.2)	0.140	68.6 (15.7)	77.1 (24.3)	0.482	69.3 (19.2)	79.7 (16.1)	**0.001**
WASH	83.1 (10.8)	80.9 (14.1)	0.385	78.4 (12.6)	81.8 (10.3)	0.493	73.2 (13.4)	66.1 (18.7)	0.386	81.0 (11.8)	79.3 (14.7)	0.424
Overall mean centre provider KAP score	69.0 (9.1)	76.0 (9.2)	**0.001**	79.6 (9.6)	83.7 (6.3)	0.158	74.9 (7.1)	70.8 (16.2)	0.588	71.7 (9.8)	76.8 (10.3)	**0.005**

### Sociodemographic characteristics of the CHVs

Of the 20 CHVs who were trained for supportive supervision, 17 participated in the intervention and were interviewed in the baseline and endline surveys, hence formed our panel data for analysis. More than half of the CHVs were from Viwandani (59%). Their mean age was 48 (SD = 6) and most (88%) were female. A majority (71%) had at least secondary education.

### CHV knowledge and practices scores

The 17 CHVs were assessed using the KAP tool, which span across eight domains. Overall, there was no significant changes in the level of knowledge and practices. Of the eight domains, only one showed a significant (positive) change, i.e., providing support supervision domain (from 78% to 94%; *p* = 0.002) (Table 7).

**Table 7:** Baseline and endline CHV KAP scores.

CHV knowledge and practice scores									
Location	Korogocho (*N* = 10) Mean (SD)			Viwandani (*N* = 10) Mean (SD)			Total (*N* = 20) Mean (SD)		
	Baseline	Endline	*p*-value	Baseline	Endline	*p*-value	Baseline	Endline	*p*-value
Learning through play	100.0 (0.0)	100.0 (0.0)	NA	100.0 (0.0)	100.0 (0.0)	NA	100.0 (0.0)	100.0 (0.0)	NA
Child protection/responsive caregiving	78.6 (26.7)	78.6 (26.7)	1.000	85.0 (24.2)	95.0 (15.8)	0.168	82.4 (24.6)	88.2 (21.9)	0.332
Communication with child	97.6 (6.3)	100.0 (0.0)	0.356	61.7 (36.0)	63.3 (15.3)	0.909	76.5 (32.8)	78.4 (21.9)	0.814
Child nutrition	100.0 (0.0)	100.0 (0.0)	NA	100.0 (0.0)	100.0 (0.0)	NA	100.0 (0.0)	100.0 (0.0)	NA
Child health	89.3 (15.2)	92.9 (14.2)	0.569	91.3 (11.9)	100.0 (0.0)	**0.045**	90.4 (12.9)	97.1 (9.4)	0.058
WASH	97.6 (6.3)	88.1 (8.1)	**0.030**	96.7 (7.0)	100.0 (0.0)	0.168	97.1 (6.5)	95.1 (7.8)	0.431
Providing support supervision	82.1 (12.2)	96.4 (9.4)	**0.030**	75.0 (23.6)	92.5 (12.1)	**0.025**	77.9 (19.5)	94.1 (10.9)	**0.002**
Attitude and perceived competence to provide support	100.0 (0.0)	90.5 (16.3)	0.172	100.0 (0.0)	100.0 (0.0)	NA	100.0 (0.0)	96.1 (11.1)	0.164
Overall mean CHV KAP score	93.1 (3.4)	93.5 (4.1)	0.838	87.0 (6.6)	92.1 (3.8)	0.0862	89.5 (6.2)	92.7 (3.9)	0.098

### Feasibility and acceptability of the CoP intervention

Feasibility and acceptability was assessed quantitatively based on the number of CHVs that were effectively trained and conducted the CoP sessions and follow up visits, and the number of centre providers who successfully completed all the modules. This was with reference to the target numbers. As shown in Table 8, we achieved the target numbers of CHVs and centre providers and for both groups of participants, we achieved the planned training/sessions and follow up visits in Korogocho and Viwandani. A total of 20 CHVs (10 from Korogocho; and 10 from Viwandani) were recruited and successfully trained on delivering the intervention. They attended all the four modules that were offered. All 10 CHVs from Viwandani facilitated one centre provider group each leading four CoP modules. In Korogocho, there were fewer centres (*n* = 16) that were eligible for inclusion in the intervention, so we purposefully used only 6 out of the 10 CHVs to lead the CoP sessions. The remaining four were put on waiting list to help in case any of the 6 became unavailable.

**Table 8:** Feasibility and acceptability indicators of the intervention.

Activity	Target number	Number (%) achieved
CHVs recruited	20	20 (100)
CHVs trained	20	20 (100)
CHVs delivering the intervention	16	16 (100)
Childcare centre/providers eligible	68	68 (100)
Childcare centre/providers recruited	68	68 (100)
CoP groups	13	13 (100)
CoP group sessions	52 (4 per group)	52 (100)
Centre providers who attended all sessions	68	58
Two follow up visits to each centre provider	136	136 (100)

A total of 68 centre providers (16 from Korogocho; and 52 from Viwandani) were eligible for the intervention, and all accepted the invite to participate. These were grouped in groups of 5–6 centre providers for the CoP sessions. Each group was managed by a CHV in charge and one back up CHV. This resulted in three groups in Korogocho and 10 groups in Viwandani. Attendance records show that all 68 centre providers attended the four modular CoP intervention facilitated by the CHVs. 117 CoP sessions altogether were conducted by the CHVs and observed by the CHAs including 27 in Korogocho, and 90 in Viwandani. Hence a total of 136 follow up visits were successfully made by the CHVs.

### Costs involved in delivering the intervention

The venue was rented at KSh 3,000/day for 9-day training in two occasions. The breakfast and lunch were provided at KSh 800/person-day for training and KSh 700/person-day for CoP sessions. The training programme was delivered by trainers from Kidogo and costed KSh 25,000/person-day for 20 person-days. The allowance paid to the CHVs and CHAs were KSh 1,000 per day and KSh 2000 per day for training and CoP sessions, respectively. The allowance paid to the CHVs for follow-up visits was KSh 700 per visit. A payment of KSh 200 per session was also given to centre providers to cover the expenses of arranging extra personnel when they attended CoP sessions, if applicable. Twenty CHVs and eight CHAs attended the training. Including necessary printing costs, the explicit costs of organizing and attending the training were estimated at KSh 1,094,100 (USD 9,979). Ninety-seven CoP sessions were delivered by two CHVs with one CHA and twenty were delivered by one CHV and one CHA. The payment for cover arrangement was given out on three occasions for each centre provider. The explicit costs of CoP sessions were therefore KSh 1,271,300 (USD 11,595). Each centre provider received two follow-up visits, costed at KSh 95,200 (USD 868). In addition, 16 CHVs and 2 CHAs were paid allowance for their activities in initial mobilization and CoP session organizing. This explicit administration cost was estimated at KSh 17,000 (USD 155).

To estimate the opportunity costs of time for CHVs and CHAs, the CHAs were costed at KSh 65,000 per month and the CHVs at KSh 3,500 per month, the latter of which had been proposed but yet to come in effect over the implementation period. The partially recorded CoP sessions indicated a median duration of 3.83 h/session (IQR: 3.35, 4.56) in Korogocho and 3.00 h/session (IQR: 3.00, 4.00) in Viwandani. The median duration of follow-up visits was 2.00 h/visit (IQR: 1.00, 2.00) in Korogocho and 1.34 h/visit (IQR: 1.00, 2.00). The opportunity costs of the CHAs were estimated at KSh 212,727 (USD 1,940) for attending training, KSh 137,940 (USD 1,258) for CoP sessions [IQR KSh 133,079 (USD 1,214), KSh 178,408 (USD 1,627)], and KSh 616 (USD 6) for administration. The estimated opportunity costs of the CHVs were KSh 28,636 (USD 261) for attending training, KSh 13,662 (USD 125) for CoP sessions [IQR KSh 13,138 (USD 120), KSh 17,622 (USD 161)], KSh 4,048 (USD 37) for follow-up visits [IQR KSh 2,705 (USD 25), KSh 5,409 (USD 49)], and KSh 530 (USD 5) for administration.

The total explicit costs were KSh 2,477,600 (USD 22,598) and the total opportunity costs were KSh 398,159 (USD 3,632) [IQR KSh 391,432 (USD 3,570), KSh 443,949 (USD 4,049)].

### Benefits of the CoP intervention on the centre provider and CHV KAP, and centre quality

Within this feasibility study, we also measured the potential benefits of the intervention on the key outcomes, i.e., centre quality, and the knowledge and practices of both CHVs and centre providers. As shown in Tables 5–7 there were generally improvements in centre provider KAP, CHV KAP and centre quality scores from baseline to endline.

As shown in Table 6, between baseline and endline, centre provider KAP score improved from 72% to 77% (*p* = 0.005). Crude and adjusted analyses revealed a significant positive effect of the intervention on centre providers’ knowledge and practices (Table 9). Without adding the confounders (unadjusted analysis), the centre providers’ mean KAP score was 0.52 standard deviations (SD) higher at post-intervention than at pre-intervention (effect size = 0.52; 95% CI: 0.17, 0.86). After adjusting for the other factors including type of centre, centre provider age, centre provider highest education, location of the childcare centre, period of operation, and centre provider ECD training, the mean centre providers’ KAP score at post-intervention was 0.47 SD higher than that at pre-intervention (effect size = 0.47; 95% CI: 0.14, 0.80). This implies that the CoP intervention significantly improved centre providers’ knowledge and practice. As was shown in Table 5, there was a significant benefits of the intervention on the quality of childcare centre quality overall score from 59% at baseline to 66% at endline (*p* = 0.002). Without adjusting for confounders, the mean quality score following 5 months’ implementation of the intervention was 0.55 SD higher than at pre-intervention (effect size = 0.55; 95% CI: 0.19, 0.91). Adjusting for the same confounders mentioned above, the mean quality of childcare score was 0.56 SD higher at post-intervention than at pre-intervention (effect size = 0.56; 95% CI: 0.19, 0.92). In other words, the CoP intervention significantly improved the quality of childcare centres. The CoP intervention did not have a significant effect on knowledge and practices of the CHVs (baseline overall score = 100 vs. endline overall score 96.1; *p* = 0.164). Of the eight domains, only one showed a significant (positive) change, i.e., providing support supervision domain (from 78% to 94%; *p* = 0.002) (Table 7). There was no significant change in overall score in either the unadjusted or adjusted analyses (Table 9).

**Table 9:** Benefits of the intervention on centre provider and CHV knowledge, practices and childcare quality.

Outcomes	Crude analysis		Adjusted analysis	
	Effect size	95% CI	Effect size	95% CI
Centre provider KAP^a^				
Pre-intervention	Ref.		Ref.	
Post-intervention	0.52^**^	[0.17, 0.86]	0.47^**^	[0.14, 0.80]
Observations^c^	116		116	
Quality of childcare centre^a^				
Pre-intervention	Ref.		Ref.	
Post-intervention	0.55^**^	[0.19, 0.91]	0.56^**^	[0.19, 0.92]
Observations	116		116	
CHV KAP^b^				
Pre-intervention	Ref.		Ref.	
Post-intervention	0.52	[−0.03, 1.07]	0.31	[−0.51, 1.13]
Observations^d^	34		34	

Reporting effect size and 95% confidence intervals in brackets. ^*^p < 0.05; ^**^p < 0.01.

a In the adjusted analysis, the model was adjusted for: type of centre (home-based, centre-based, faith-based), centre provider age (years), centre provider highest education (primary, secondary, tertiary), location of the childcare centre (Korogocho, Viwandani), period of operation (years), and centre provider ECD training (yes, no).

b In the adjusted analysis, the model was adjusted for: CHV age (years), CHV sex (female, male), CHV highest education level (primary, secondary, tertiary), and CHV area of operation (Korogocho, Viwandani).

c Observations represent the number observed at baseline (*n* = 58) and at endline (*n* = 58), totalling to 116 centre providers/childcare centres.

d Observations represent the number observed at baseline (*n* = 17) and at endline (*n* = 17), totalling to 34 CHVs.

### Qualitative findings

The qualitative findings contributed to our understanding of changes in knowledge and practices in relation to the four main topics of the intervention. Table 10 below shows the level of agreement, discordance and silence across the quantitative and qualitative data.

**Table 10:** Meta inferences by intervention domain.

	Quantitative: significant improvements	Qualitative: findings from interviews, focus groups, observations across all respondents	Meta-inference
(i) Learning through play.	Increases in the proportion of centres with toys for children to play with, driven by home-based centre improvements. Remained similar in small centres and faith-based organisations.	Providers and parents gave examples of centres promoting play and providers making and using toys made from local materials as shown during CoP meetings.	Confirmatory findings: although providers felt the need for more toys and guidance.
(ii) Safety and security, child safeguarding.	Overall improvement in child protection, safety, abuse and positive discipline, driven by improvements in home-based centres.	Limited information in the qualitative data.	Silence: respondents in the qualitative methods did not identify child safety and security as a particular area of change
(iii) Essential child health, sanitation, hygiene and nutrition	Health: Improvements in knowledge of immunisation schedule, driven by home-based centres. Hygiene: Limited change in hygiene practices. Nutrition: Increases in the proportion of centres overall serving hot food, driven by home-based centres.	In addition to knowledge of immunisation, parents, providers and CHVs mentioned greater knowledge among providers on responding to a sick child. *“Immunization you know when you take your child to her childcare you – I have told you she writes down your name and phone number and your partner if you have one and the child’s name and the age, so she can know if the child has completed their immunization or not. If the child has completed, she doesn’t follow up but if not yet immunized she reminds you, this child is this age he/she needs to be taken for this immunization.”* (Parent 008-Viwandani) “*if they [centre-providers] realise that a child health is not good they refer them or they call me to refer them and things like these, and they also have some skills in case the child feel sick suddenly they leave their work and know how they will help the child to get to the hospital.”* (CHV 001-Viwandani) *“Mine [child] got sick and when she [centre provider] saw the child isn’t well, she called me and told me that she[child] was sleeping and she was complaining of a headache. She [centre provider] asked if I have any medicine I can bring. I told her I didn’t have medicine, and that it it’s better I come and take her to the hospital. So, she knows how to look after the children she can know a child who has a problem or not.”* (Parent 010-Korogocho) Implementation of handwashing with soap observed in some centres: *“I have seen a difference in how the children are washing their hands there; it’s not like how you would take your child to wash their hands so they can go back to eat. I saw that she has put equipment for children to wash their hands with soap.”* (Parent 002-Viwandani) Participants reported how providers now emphasised the need for a balanced diet, with vegetables and fruit. “*You find they [centre providers] tell the parents to buy various fruits for the children. So, you find oranges, bananas in this childcare centre and they weren’t there [before].”* (CHV 010-Viwandani) *“I have seen there are some changes because before I was taking my child maybe with porridge, food but not fruits because sometimes I would not be able to afford fruits and she asked me when I went to pick up my child, she told me that it is good to mix food and some fruits for the child even if I’m struggling.”* (Parent 008-Viwandani)	Agreement and extension: qualitative data highlighted greater knowledge and response to sick children. This may not have been identified in the questionnaire due to limited incidence of child sickness. Discordance: questionnaire did not confirm qualitative responses on improved hygiene. Discordance: qualitative data was silent on hot food. Questionnaire did not capture the resource challenges in implementing nutritional changes.
(iv) Business management	Improvements in registration, policies, finance tracking and budgeting overall, driven by home-based centres.	Centre providers valued business training and tracking expenditure, leading to a recalibration of fees for some. Providers and CHVs emphasised difficulties in collecting fees and manage their business when parents are struggling financially, working long and unpredictable hours.	Confirmation with caveats: while business management skills improved following the intervention, implementing regular fee collection and ensuring fees cover expenditure is extremely challenging within the informal work context of these families.

In addition to the insights on the impacts of the training and CoP sessions on the four areas of learning through play, safety, health and business management, the qualitative data provided further insights on the implementation and impact of the intervention. These included: (i) the influence of the slum context; (ii) the changing roles of CHVs, childcare centres and providers and; (iii) the need to extend the reach of the intervention. Quotes relating to these themes are provided in the Annex section.

#### Struggling to improve within the context of informal settlements

Despite the improvements highlighted in both the questionnaire and the qualitative findings, the context of the informal settlements continually undermined the ability of childcare centre providers to implement their new skills and improve their practice. This was seen across the areas covered in the CoP sessions. For example, while there was convergent evidence across qualitative respondents and quantitative results regarding nutrition knowledge and practices, the economic challenges facing families and providers undermined the possibility of adding additional fruit, vegetables and greater variety of food in the centres.

The quantitative findings indicated how the centre providers, particularly those running home-based centres learnt how to manage the finances and running of their centres. Others however, explained how the business training had helped them to decide on a fee scale that would cover their costs, although they felt unable to increase the price for existing parents, who were already struggling to pay the existing charges. This may explain why none of the parents interviewed complained of fee increases. While parents were still unhappy at the poor physical structures of the childcare centres, both providers and parents shared examples of centres being reorganised to promote play and providers making and using toys they made themselves from local materials as practiced in the CoP sessions. In a few of the centres, CHVs reported inconsistences in the centre providers that attended the sessions. Frequent changes of the centre providers or assistants interfered with the content that was given to the providers, and the overall benefit (knowledge and skills improvement) in that particular centre. So the CHVs felt that such childcare centres did not fully benefit from the sessions because different centre providers were trained on different sessions.

#### Changing roles for CHVs, childcare centres and providers

While all the CHV’s had been active in their communities, many explained how systematically visiting childcare centres and supporting them to improve child health and development had not been part of their previous routine work. All CHVs interviewed expressed their enthusiasm for the new role. This change in the role of CHVs clearly took a while to become established and accepted by the childcare providers, particularly those in home-based centres who were not used to receiving any support, as shown in the quantitative results. The observations of the CoP sessions and the interviews with providers highlighted their enthusiasm for the knowledge and skills that they gained throughout the intervention. Parents were frequently encouraged by the increased competency of the childcare providers and several commented on ways that they could be further trained and supported. This was particularly true in relation to health, both in the provision of medicines for minor illnesses by the providers themselves or by taking the child to hospital if needed. Parents frequently talked about the challenges they faced in accessing health care for their children due to their long working hours and long commute journeys. Many felt that the childcare centres could usefully become a point of access for child health programs including immunizations.

#### Acceptability and the need to extend the reach of the programme

The high number of private and informal childcare centres within the slums and the ease with which inexperienced providers can open a centre was recognised by all participant groups. CHVs were enthusiastic about the project and felt that CHVs in other areas could be trained to deliver the programme in their own catchment areas as part of their routine activities which further indicated acceptability of the intervention.

## Discussion

The current study aimed to evaluate the feasibility acceptability, cost and potential benefits of a community of practice (CoP) model for improving the quality of informal childcare services in two informal settlements in Nairobi. Quantitative and qualitative findings revealed strong indicators of feasibility and acceptability of the intervention among various stakeholders including parents, CHVs, centre providers, policy makers at the National, County and sub-county level in health and education sectors. The findings clearly show that it is feasible to train CHVs to deliver training and support supervision of centre providers on provision of quality childcare services when the content is tailored to their level of education and embedded in their routine work. The data also show a potential for the intervention to improve centre provider knowledge and practices, and the quality of the childcare centre environment.

The feasibility and acceptability of the CoP intervention is attributed to the community participatory approach that was used from the co-design stage, through implementation to evaluation of the model. We involved government representatives, non-government stakeholders (e.g., Kidogo), and the local community (CHVs, centre providers and parents) first to identify the gaps in quality, the issues facing informal childcare and ideas on how they can be addressed. We jointly co-designed the model and CHVs were identified to deliver it. We noted and applied the learning during the implementation and at the end, we jointly refined the model and put together a manual describing the content and its delivery. Participating in and contributing to the development and implementation of the intervention promoted buy-in and sense of ownership among various stakeholders, and made it easy for the research team to obtain genuine feedback on how the intervention can be optimized. Community involvement in health programmes has far reaching benefits in promoting community programmes (37–40) and for the CoP intervention, it will enable full integration, sustainability and transition to scale planned in the future.

The success of CHV and centre provider training sessions was majorly a result of how they were administered – spacing the topics a month apart to allow internalization and application, but also utilizing the interactive approach and taking into account the low literacy and socio-economic levels of both the CHVs and centre providers. Administering the sessions in the local language and use of simple tools made the sessions easy to administer and follow up to be done.

It is important to note also that effective engagement of CHVs in community programmes requires that they are incentivized. In this study, CHVs were given transport reimbursement and a day’s allowance whenever they engaged in the programme activities. Despite that it was a small allowance it contributed significantly to their motivation and willingness to deliver the intervention. The role of community health workers or volunteers in supporting health care programmes in communities is increasingly being recognized. There has been a lot of debate on how CHVs can be fairly remunerated for the work they do and how they can be motivated to continue supporting community initiatives. Our findings are consistent with those from other low income settings where incentives including transport reimbursement, recognitions and stipends resulted in significant motivation for CHVs to support community health programmes (41, 42). In Kenya, for example, transport was considered more incentivizing than tools of trade and the monthly stipend. Specifically, CHVs preferred job incentives that offered higher monthly stipends, and recognition at community level over award mechanisms for the best performing CHVs (42). On the contrary, in Indonesia, CHVs were happy with a small monthly financial benefit and were more motivated by enhanced methods of performance feedback, training and considerations for their rights and responsibilities. These and other contextually appropriate incentives may need to be considered when integrating the CoP intervention into CHVs’ routine work for optimum delivery (41).

We leveraged on the existing infrastructure, particularly the CHV system to deliver the programme. The CHVs were supported and supervised by the community health assistants (CHAs) and nutritionists as was the practice within the community health strategy at no extra cost, apart from the transport reimbursement when CHAs attended meetings. Costing data revealed that the explicit cost of the entire intervention was 22,589 USD, of which 41% (9,181 USD) were allowances paid to CHVs and CHAs for training and intervention activities. In contrast, the estimated opportunity cost of time of CHVs and CHAs were only 3,632 USD. It poses questions for decision makers to consider: is the incentive sustainable outside of the study setting, given the budget? For scale-up of the CoP intervention and its integration into the routine practice, a balance must be struck in the long run, between reduced or even removal of incentives and increased basic salary for CHVs. At the higher level, decision makers at the national and County level (Ministry of Health, and Education) were continually consulted and their approval to use the sub-county teams to deliver the programme was obtained. Imbedding the intervention in the existing system made implementation cheaper and easier and hence contributed to its feasibility. Research has shown that successful community programmes are those that have utilized existing infrastructure (43). The integrated approach promoted ownership, enabled resource mobilization, minimized costs of delivery, and to some extent provided initial capacity which are altogether critical for the sustainability of the programme in a resource limited health system. The delivery approach used in this study meets the recommendations that any integrated service delivery model should be developed after a formative research conducted with the users, the providers, and the existing physical and functional system for providing the service; and that it should be planned through a participatory process with involvement of all cadres of stakeholders including senior health officials and bureaucrats at the top to the end users at the bottom (43). The team of centre providers, CHVs, and their supervisors provide a critical capacity (champions) to cascade the acquired knowledge and practices to the rest of the community and to therefore enable implementation to scale. The intervention has the advantage to be adopted to other settings that have the CHV system or its equivalent.

In addition to the evidence on the acceptability and feasibility of the CoP intervention, our findings (both quantitative and qualitative) also indicate a potential of the intervention to improve the knowledge and practices of centre providers as well as the quality of the centre environment. It is important to note that home-based centres had the worst level of quality, and these appeared to be a major driver of mean score on quality. Home-based centres which characterised the majority of centres in Korogocho, showed marked improvements in quality after the intervention revealing a differential benefit of the intervention for this type of centres. The obvious explanation is that the majority of these were of poorer quality at the start and were not receiving any form of support or training prior to the intervention and hence they need this training most. The finding indicates that future interventions should prioritise home-based centres. Absence of changes in CHVs’ knowledge scores was most likely because they already had sufficient knowledge at baseline and therefore there was limited window for a significant change attributable to the training.

It should be noted that this was a feasibility study for which sample size was not powered to measure effects of the intervention, however, the fact that it shows indicators for the benefits points to its potential to improve childcare in informal settlements. Full impact of the intervention can be established using larger studies with well powered sample sizes and longer follow up.

It should be noted that, while formal, well-equipped centre-based care with appropriate facilities and adequate numbers of ECD-trained staff may be seen as the ideal, without major investment and subsidization, such provision within informal settlements is unlikely, particularly in the short-term. There is increasing recognition of the dynamism and entrepreneurial spirit found within informal settlements, challenging a rethink of simplistic dichotomies where the ‘informal’ is seen as wholly negative and in contrast to formal services (44). This came out clearly through discussions with the different stakeholders. Supporting and enhancing these community-based informal childcare centres through a model like this one, offers opportunities for not only improving child-health, ECD and the livelihoods of working parents, but also building economic opportunities for childcare providers and potentially strengthening social capital within the often transient informal settlements. Due to rapid urbanization, women in urban informal settings work outside the home frequently for long-hours and in unstable informal jobs. They no longer have the support of the extended family that they may have relied on in rural areas to provide care for their children. With an estimated 89%–95% of women working in the informal sector in in Sub-Saharan Africa (45), there is an urgent need for childcare solutions for these women (46). The CoP model therefore provides a promising solution to the challenges faced by urban-poor families in providing a safe, nurturing and healthy environment for their under-5 children.

In conclusion, it can be very challenging for providers to improve their centres within the context of informal settlements. However, change is possible, particularly for the least supported and poor quality home-based centres. This highlights the potential of an affordable intervention, grounded within the existing public community health system to improve the quality of childcare centres, holding out the hope for improvements in child health and development outcomes. The current study provides insightful evidence on how a participatory approach can be effectively used to design and deliver an intervention to improve informal childcare services in a resource limited setting. To our knowledge, this model is the first of its kind and its potential lies in the co-design, community involvement and integration approach that was used in its development and delivery. The evidence on its feasibility and acceptability from the community as well as the indicators for potential impact provide a promise for an impact evaluation study including its benefits for child outcomes which will in turn inform further evaluation of its impact at scale. It would be useful for future studies to explore a multi-sectoral approach to implementation of the intervention by strengthening the links with similar initiatives across sectors to avoid parallel systems but also to ensure sustained funding. The implementation manual, detailing the topics and delivery, is a valuable product that can be used to support wider implementation in Kenya and could be adopted or adapted to other low income settings. Future studies exploring and addressing the barriers to regulation and licensing of informal childcare centres are needed since it was clear from the data that the majority of the childcare were neither registered/licensed nor receiving any support from the government. Lack of clarity of the regulations as well as unachievable expected standards and high fees are major barriers to registration of informal childcare centres.

### Limitations

While we had to collect some data virtually, this may have limited the depth of discussion and reflections. We attempted to mitigate these limitations by doing spot checks by going back to randomly selected households that were interviewed by the field team and asking a sample of the questions to compare the agreement of the responses.

There are very large variations in the quality scores according to the type of centre at baseline and some scores (e.g., within faith-based centres) decrease between baseline and endline. It is not clear why but it might be that centre providers focused on certain areas at the cost of other aspects. The variations probably had some implications to overall findings presented.

The current study primarily aimed to assess the feasibility of a co-designed CoP model, but not powered to assess impact on centre provider KAP or quality of environment, or child outcomes. Hence the data reported here only provide an indication for possible benefit of the intervention. For the same reason, we costed the intervention however, it would not be appropriate to examine cost effectiveness without robust effects data in terms of improvement of quality or child outcomes (not measured). Future studies designed to rigorously evaluate impact on centre quality and child outcomes as well as cost effectiveness are underway.

## Data availability statement

The raw data supporting the conclusions of this article will be made available by the authors, without undue reservation.

## Ethics statement

The studies involving human participants were reviewed and approved by Amref Health Africa’s Ethics and Scientific Review Committee ESRC, Kenya (Ref: P7802020 on April 20, 2020) and from the University of York (Ref: HSRGC March 20, 2020). The participants provided their written informed consent to participate in this study.

## Author contributions

MN co-led the acquisition of funding, conceptualization, investigation, and methodology, led the preparation of the manuscript, project administration and supervision, and participated in data curation and formal analysis, review and editing of the manuscript. NL led data analysis and curation, and participated in project administration, and editing of the manuscript. LO participated in the project administration, data curation, formal analysis, and writing. KO participated in the conceptualization of the investigation and methodology of the study, project administration, data curation, formal analysis, and writing. PA participated in project administration, data visualization, and editing of the manuscript. RM, SH, and MK participated in project administration, data visualization, and writing. AR and MA-O participated in project administration, data curation and visualization, and writing of the manuscript. PK-W participated in the conceptualization, investigation and methodology, project administration, data curation and visualization, and writing. EK-M contributed to the design and supported the administration of the project, and writing. HE led funding acquisition, conceptualization, investigation, and methodology, co-led project administration and supervision, and formal analysis and manuscript writing. All authors contributed to the article and approved the submitted version.

## Conflict of interest

SH and MK were employed by Kidogo Innovations, Nairobi, Kenya.

The remaining authors declare that the research was conducted in the absence of any commercial or financial relationships that could be construed as a potential conflict of interest.

## Publisher’s note

All claims expressed in this article are solely those of the authors and do not necessarily represent those of their affiliated organizations, or those of the publisher, the editors and the reviewers. Any product that may be evaluated in this article, or claim that may be made by its manufacturer, is not guaranteed or endorsed by the publisher.

## Annex Selected quotes relating to the themes in the qualitative results

### Struggling to improve within the context of informal settlements

On the side of food, when I was doing my assessment, I saw that people are still in down… some day-care centres were getting rice but it is just pure white rice which is not mixed with anything and it is just very dry. You see that is the rice they are eating and when I engage her she tells me that is what they can afford. (CHV 007-Korogocho)

Like the ones who are joining now, you know the ones I had before I can’t charge them more, like now how we have agreed as daycare providers is we should charge the ones joining more. (Provider 010-Viwandani)

Some don’t pay. They bring a child and they say they will pay you. [But] they run away and go somewhere else …they bring a child without food and you can’t stay with that child without food, you will have to use your money to buy that child food or you give them what you are eating because so many do this. (CHV 004 – Viwandani)

Even the playing items for children; we didn’t have them before, we didn’t know we can find them around and they are important because we couldn’t afford to buy them but after the training now, we made ours from what we can find and now they play with them. (Provider 008-Korogocho)

We did not have play materials; in fact, we did not even know the importance of play as part of learning. But we were sown to make play materials from available things like tissue rolls. But if we can get more, it can be good because the children will not be fighting for the few that we make. Each child will have a toy to play with. (Centre provider Viwandani)

It was hard at first because you know, let's say I have a childcare and I don’t have someone else to leave with these children and I am coming for the session. So, you see she is wondering how she will leave these children and go to a session. So, it was a little hard but after a while they started getting someone to sit in but they also had stress because if a centre provider is used to this child, the one she has left may not understand the mood of the child and feeding such a child for example becomes difficult. (CHV 004-Viwandani)

Another challenge that I faced in one childcare is that the provider loves changing the teachers, so you were training a certain person then the next day another, so that is a challenge, but it only happened in one childcare. (CHV001 – Viwandani)

### Changing roles for CHVs, childcare centres and providers

Before I was going every month as I had told you and I wasn’t visiting all of them before we started this project. But nowadays I have to set aside a day to visit my childcares one by one as I know this has this problem and we solve this issue. (CHV 002-Viwandani)

Those childcare centre providers when I went to talk to them, they were scared because they were wondering who these people are. We explained to them and told them that these people are in the community and they are coming to enlarge their training on ECD. They just said it was okay although the turn up was not good on the first day but the second day they came in a good number. (CHV 005-Viwandani)

The first aid training was very good. They even demonstrated for us. They taught us how to handle cases that we didn’t know how to deal with. They empowered us very much. We can do very well if we can be provided with full first aid kits. (Centre provider 009-Korogocho)

You find so many people here and if you get a job in a company, you even forget to take your child to the clinic. [If] the children could be immunized in the centre because this will save time for the parents who are busy at work. (Parent 008-Viwandani)

### The need to extend the reach of the programme

Now you can’t find anyone employing a house help or a child who is being left with a neighbour… When you take your child there [supported childcare centres] you will feel safe so, it would be good if the childcares left out could be included so they can get what the others got. (CHV 002-Viwandani)

So my humble appeal is that if it is possible it can be rolled out to even the other childcare centres that were not included. (CHV 007-Korogocho)

It is a good idea since there are some who are starting these baby cares and they don’t have the skills. You get they leave the children to get cold but when they have this information on how to take care of children at least when we go to work as parents, we will feel our children are in safe hands. So, it is good to empower them how to take care of children. (Parent CP005-Korogocho)
