# Supplementary material for: The feasibility, acceptability, cost and benefits of a “communities of practice” model for improving the quality of childcare centres: a mixed-methods study in the informal settlements in Nairobi
Source: Front Public Health. 2023 Aug 1;11:1194978. doi: 10.3389/fpubh.2023.1194978 (PMC10426909; doi:10.3389/fpubh.2023.1194978)
Supplement: Supplementary file 1 [file Table_1.docx]

**APPENDICES**

**Appendix 1: Baseline characteristics of childcare center providers in the baseline-endline panel and those not in the panel**

| **Variable** | **Level/statistic** | **In panel** | **Not in panel** | **p-value** |
| --- | --- | --- | --- | --- |
|  |  | **N=58** | **N=8** |  |
| Location of centre | Korogocho | 15 (26%) | 0 (0%) | 0.180 |
|  | Viwandani | 43 (74%) | 8 (100%) |  |
| Center provider age (years) | Mean (±SD) | 40.2 (±9.0) | 40.3 (±16.2) | 0.980 |
| Center provider sex | Male | 3 (5%) | 0 (0%) | 1.000 |
|  | Female | 55 (95%) | 8 (100%) |  |
| Center provider highest education level | None | 2 (3%) | 1 (13%) | 0.230 |
|  | Primary | 25 (43%) | 2 (25%) |  |
|  | Secondary | 22 (38%) | 5 (63%) |  |
|  | Tertiary | 9 (16%) | 0 (0%) |  |
| Center quality score | Mean (±SD) | 59.2 (±11.6) | 53.6 (±8.4) | 0.200 |
| Center provider KAP score | Mean (±SD) | 71.7 (±9.8) | 70.2 (±9.5) | 0.670 |

**Appendix 2: Quality of daycare center (center environment) scoring guide**

| **Component** | **Question** | **Responses** | **Score of response** | |
| --- | --- | --- | --- | --- |
|  |  |  | **0** | **1** |
| **Child protection, Child safety, Child abuse, & positive discipline**  **Stimulating Environment** | Safe & free of obvious hazards (e.g. sharps, fire, ditches, live wires, collapsing walls, hot water, medicines, small beads/beans) | 1-Yes  2-No | 2 | 1 |
|  | Sufficient lighting & ventilation (room not dark) |  |  |  |
|  | At least one mat / carpet |  |  |  |
|  | Suitable indoor space for the number of children (one child per sq. Meter) |  |  |  |
|  | Children look happy and calm (e.g. not looking irritable, grumpy , crying, nervous, or/and depressed) |  |  |  |
|  | Comfortable outdoor space (spacious, not crowded, dry, and flat) |  |  |  |
|  | No instance of corporal punishment observed (i.e. yelling, hitting, pinching, other physical punishment) |  |  |  |
| ***Maximum component raw score = 7; Percent score = (Raw score/7)*100%*** | | | | |
| **Responsive Caregivers** | Fifteen children (or less) per caregiver | 1-Yes  2-No | 2 | 1 |
|  | Caregiver is aware of all children and attends to their needs(knows about each child individually and their needs) |  |  |  |
| ***Maximum component raw score = 2; Percent score = (Raw score/2)*100%*** | | | | |
| **Learning through Play** | Each child has something to play with | 1-Yes  2-No | 2 | 1 |
|  | Daily routine posted & used |  |  |  |
|  | One learning centre present & labelled (separate area with play materials, toys, books etc. for learning) |  |  |  |
| ***Maximum component raw score = 3; Percent score = (Raw score/3)*100%*** | | | | |
| **Health** | Conducts daily health check and understands what to do if a child is sick | 1-Yes  2-No | 2 | 1 |
|  | First aid kit & knowledge on use |  |  |  |
|  | Immunization status of children tracked |  |  |  |
|  | Evidence for temperature recording (thermometer, temp records available) |  |  |  |
|  | ***Evidence for protection against Covid 19:*** |  |  |  |
|  | Handwashing station (clean water and soap) present |  |  |  |
|  | Sanitizer present |  |  |  |
|  | Masks present |  |  |  |
| ***Maximum component raw score = 7; Percent score = (Raw score/7)*100%*** | | | | |
| **Nutrition** | Children receive morning *uji* (porridge) | 1-Yes  2-No | 2 | 1 |
|  | Children receive lunch |  |  |  |
|  | Children are served with warm food |  |  |  |
|  | Poster of what a balanced diet is, is displayed |  |  |  |
| ***Maximum component raw score = 4; Percent score = (Raw score/4)*100%*** | | | | |
| **Water, Sanitation & Hygiene (WASH)** | Hand washing facility with soap available and in use | 1-Yes  2-No | 2 | 1 |
|  | At least one potty for every 5 children |  |  |  |
|  | Centre is cleaned daily (centre is visibly clean) |  |  |  |
|  | Access to clean and safe drinking water |  |  |  |
| ***Maximum component raw score = 4; Percent score = (Raw score/4)*100%*** | | | | |
| **Business & Administration** | Daily attendance recording available | 1-Yes  2-No | 2 | 1 |
|  | Track finances daily/ weekly / monthly (record available) |  |  |  |
|  | Centre policies clearly posted(any information on how their centre is run/managed) |  |  |  |
|  | Budget available |  |  |  |
| ***Maximum component raw score = 4; Percent score = (Raw score/4)*100%*** | | | | |
|  | | | | |
| **Maximum total center quality raw score = 7+2+3+7+4+4+4 = 31;**  **Total center quality percent score = Mean of component percent scores (sum of all component percent scores/7)** | | | | |

**Appendix 3: Centre caregiver KAPs scoring guide**

| **Domain** | **Question** | **Responses** | **Scoring of response** | |
| --- | --- | --- | --- | --- |
|  |  |  | **0** | **1** |
| **Knowledge on Business management** | It is important to develop the budget for the day-care centre at the beginning of the term | 1 - Agree completely 2 - Agree a little 3 - Disagree a little 4 - Disagree completely | 3,4 | 1,2 |
|  | It is important to record which parent has paid |  |  |  |
|  | It is important to record how much each parent has paid |  |  |  |
|  | Do you/centre owner track the income and expenses made in your centre? | 1-Yes  2-No | 2 | 1 |
|  | Do you make or prepare a budget for your centre at the beginning of the week/month? |  |  |  |
|  | Do you have the centre policy on pricing and opening times for the centre? (Centre name, operating hrs, fees, and caregiver contacts).  *Ask the centre provider if it is not visible* |  |  |  |
|  | Do you track/keep accounts for your centre showing income and expenditure? |  |  |  |
|  | Do you track attendance daily? |  |  |  |
|  | Is the business license posted? |  |  |  |
|  | Do you have an attendance register showing how many children come to your centre per day? |  |  |  |
| ***Maximum component raw score = 10; Percent score = (Raw score/10)*100%*** | | | | |
| **Knowledge on Safety** | It is important to always ensure a safe environment for the children | 1 - Agree completely 2 - Agree a little 3 - Disagree a little 4 - Disagree completely | 3,4 | 1,2 |
|  | It is important for a caregiver or other people to keep a child in visual range and to look at him/her often |  |  |  |
|  | Centre providers should constantly watch out for and remove any potentially hazardous materials (e.g. sharps, fire, ditches, live wires, collapsing walls, hot water, medicines, small beads/beans) |  |  |  |
| ***Maximum component raw score = 3; Percent score = (Raw score/3)*100%*** | | | | |
| **Knowledge on responsive caregiving/discipline** | Children must be handled harshly for them to develop better. | 1-Always  2-Sometimes  3-Never | 1,2 | 3 |
|  | Which method do you use most often to deal with children when they misbehave?  *(tick only one option)* | 1-Physical punishment  2-Verbal punishment  3-Distract child with another activity  4-Explains wrong deeds to child calmly | 1,2,3 | 4 |
|  | In the past 2 weeks, how many times have children been physically punished in the centre? *(pinch/slap/spanked )* | 1-Daily  2-Once to twice in a week  3-Never | 1,2 | 3 |
| ***Maximum component raw score = 3; Percent score = 3(Raw score/3)*100%*** | | | | |
| **Learning through play** | It is important for children to play | 1 - Agree completely 2 - Agree a little 3 - Disagree a little 4 - Disagree completely | 3,4 | 1,2 |
|  | Does each child get an opportunity to play with toy or something | 1-Yes  2-No | 2 | 1 |
|  | Do you post the Children’s work on the wall |  |  |  |
| ***Maximum component raw score = 3; Percent score = (Raw score/3)*100%*** | | | | |
| **Knowledge on health** | It is important to always take a child’s temperature on arrival at the day care | 1 - Agree completely 2 - Agree a little 3 - Disagree a little 4 - Disagree completely | 3,4 | 1,2 |
|  | Conduct daily health checks? | 1-Yes  2-No | 2 | 1 |
|  | Do you understand what to do if a child is sick? |  |  |  |
|  | Do you know if the children you care for have been immunized? | 1-Yes for all  2-Yes for some  3-No | 3 | 1,2 |
|  | Can you tell me which vaccinations children should have and when? (*Interviewer confirms if answer given by centre provider is correct or wrong*) |  |  |  |
|  | Birth (BCG & Polio 0) | 1-Correct  2-Wrong | 2 | 1 |
|  | six weeks (DPT and Polio 1) |  |  |  |
|  | Ten weeks(DPT2 and Polio 2) |  |  |  |
|  | Fourteen weeks (DPT3 and Polio 3) |  |  |  |
|  | Nine months (measles) |  |  |  |
|  | *(NB: Interviewer will have to know the immunization schedule and compare their answer to tick correct or wrong)* |  |  |  |
| ***Maximum component raw score = 9; Percent score = (Raw score/9)*100%*** | | | | |
| **Nutrition** | Children must be fed with a balanced diet to be healthy, and grow and develop well | 1 - Agree completely 2 - Agree a little 3 - Disagree a little 4 - Disagree completely | 3,4 | 1,2 |
|  | How often do you encourage and make sure children finish all the food on their plates: *(Tick only one)* | 1-Every meal  2-Often I’m too busy so can only do this sometimes  3-I never have time to do this  4-I think it is better to let children eat on their own | 2,3,4 | 1 |
|  | Do you plan and provide a menu with diverse foods for a day/week/month? | 1-Yes  2-No | 2 | 1 |
|  | If you don’t cook in your centre, do you advise parents on the foods to give their children to bring with them? |  |  |  |
|  | Do you feel confident telling them which foods are appropriate? |  |  |  |
| ***Maximum component raw score = 5; Percent score = (Raw score/5)*100%*** | | | | |
| **Water, Sanitation & Hygiene (WASH)** | When do you do handwashing in the daycare centre? *(tick or cross against each item)* |  |  |  |
|  | Before preparing meals | 1-Yes  2-No | 2 | 1 |
|  | After changing diapers |  |  |  |
|  | After handling soiled toys |  |  |  |
|  | After visiting the toilet |  |  |  |
|  | How do you do your handwashing? | 1-With water only  2-With water and soap  3-No handwashing | 1,3 | 2 |
|  | How often do you clean the centre? | 1-Once a day  2-More than once a day  3-Every other day | 1,3 | 2 |
|  | What type of drinking water do you use? | 1-Unboiled water  2-Boiled water  3-Water treated with water guard  4-Bottled water | 1 | 2,3,4 |
|  | How do you dispose of your potty/ diapers waste? | 1-In the trench  2-Toilet  3-Dustbin  4-Pit latrine  5-Hole dug | 1,2,3 | 4,5 |
| ***Maximum component raw score = 8; Percent score = (Raw score/8)*100%*** | | | | |
|  | | | | |
| **Maximum total centre caregiver KAPs raw score = 10+3+3+3+9+5+8 = 41;**  **Total centre caregiver KAPs percent score = Mean of component percent scores (sum of all component percent scores/7)** | | | | |

**Appendix 4: CHV KAPs scoring guide**

| **Domain** | **Question** | **Responses** | **Scoring of response** | |
| --- | --- | --- | --- | --- |
|  |  |  | **0** | **1** |
| **Learning through play** | Young children learn from play | 1 - Agree completely 2 - Agree a little 3 - Disagree a little 4 - Disagree completely | 3,4 | 1,2 |
|  | Centers need to provide space, toys and time for children to play |  |  |  |
| ***Maximum component raw score = 2; Percent score = (Raw score/2)*100%*** | | | | |
| **Child protection/Child safety/Abuse/positive discipline/responsive caregiving** | Children must be handled harshly for them to develop better  *(Harshly means shouting, physical beating, yelling, or any form of force used to handle children)* | 1 - Always  2 - Sometimes  3 - Never | 1,2 | 3 |
|  | How should children who misbehave be corrected?  *(Tick all that apply)* | 1 - Using Physical punishment  2 - Using Verbal punishment  3 - Distract child with another activity  4 - Explain wrong deeds to child calmly | 1,2 | 3,4 |
| ***Maximum component raw score = 2; Percent score = (Raw score/2)*100%*** | | | | |
| **Communication with child** | *The following communication/interaction practices with children are appropriate:* | 1 - Yes  2 - No | 1 | 2 |
|  | Very little talk or no conversation needed |  |  |  |
|  | Shouting at/ speaking angrily at the children/ with irritation |  |  |  |
|  | Showing no interest in children’s conversation |  |  |  |
|  | Allowing the child to express themselves freely in the conversation | 1 - Yes  2 - No | 2 | 1 |
|  | Children’s needs (e.g. if a child cries or sleepy or hungry) must be responded to:  *(Please give the options to the respondent)* | 1 - Immediately  2 - Anytime  3 - Never | 2,3 | 1 |
| ***Maximum component raw score = 5; Percent score = (Raw score/5)*100%*** | | | | |
| **Nutrition** | Children should be fed with: | 1 - Same foods everyday  2 - Variety of foods rotated on different days | 1 | 2 |
|  | It is important that children are served warm (not cold) food | 1 - Agree completely 2 - Agree a little 3 - Disagree a little 4 - Disagree completely | 3,4 | 1,2 |
|  | Caregivers should have knowledge about a balanced diet , and the different food groups |  |  |  |
| ***Maximum component raw score = 3; Percent score = (Raw score/3)*100%*** | | | | |
| **Health** | It is important to know about the immunization status of children in day cares | 1 - Agree completely 2 - Agree a little 3 - Disagree a little 4 - Disagree completely | 3,4 | 1,2 |
|  | Can you tell me which vaccinations children should have and when? (*Interviewer confirms if answer given by centre provider is correct or wrong*) | 1-Correct  2-Wrong | 2 | 1 |
|  | Birth (BCG & Polio 0) |  |  |  |
|  | six weeks (DPT and Polio 1) |  |  |  |
|  | Ten weeks(DPT2 and Polio 2) |  |  |  |
|  | Fourteen weeks (DPT3 and Polio 3) |  |  |  |
|  | Nine months (measles) |  |  |  |
|  | *(NB: Interviewer will have to know the immunization schedule and compare their answer to tick correct or wrong)* |  |  |  |
|  | There should be daily health check and an understanding of what to do if a child is sick | 1 - Agree completely 2 - Agree a little 3 - Disagree a little 4 - Disagree completely | 3,4 | 1,2 |
|  | Center providers should be knowledgeable on how to conduct first aid in case of an emergency |  |  |  |
| ***Maximum component raw score = 8; Percent score = (Raw score/8)*100%*** | | | | |
| **WASH** | When should handwashing be done? Handwashing should be done (*tick or cross against each item*) | 1 - Before preparing meals  2 - After changing diapers  3 - After handling soiled toys | - | 1,2,3 |
|  | How do you do your handwashing? | 1 - With water only  2 - With water and soap  3 - No handwashing | 1,3 | 2 |
|  | Young children need to have their hands washed with soap before feeding | 1 - Agree completely 2 - Agree a little 3 - Disagree a little 4 - Disagree completely | 3,4 | 1,2 |
|  | It is ok for young children in a day care center to share feeding utensils while they are feeding | 1 - Yes  2 - No | 1 | 2 |
| ***Maximum component raw score = 4; Percent score = (Raw score/4)*100%*** | | | | |
| **Providing support supervision** | How often do you visit child care centers | 1 - Weekly  2 - 1-3 times a monthly  3 - Less than once a month  4 - Never | 3,4 | 1,2 |
|  | How much time do you spend at the child care centers when you visit | 1 - Up to 30 mins  2 - 30 - 60 mins  3 - More than an hour | 1 | 2,3 |
|  | Do you support/advise center providers? | 1 - Yes  2 - No | 2 | 1 |
|  | How often do you support them? | 1 - Weekly  2 - Monthly  3 - Less than once a month | 3 | 1,2 |
| ***Maximum component raw score = 4; Percent score = (Raw score/4)*100%*** | | | | |
| **Attitude and perceived competence to provide support** | Do you feel motivated to support day-care centers within your role as a CHV? | 1 - Strongly motivated  2 - Motivated  3 - Somehow motivated  4 - Not motivated | 3,4 | 1,2 |
|  | Do you feel confident to support day-care providers to improve the quality of their day-care? | 1 - Yes  2 - No | 2 | 1 |
|  | Do you feel you have the required competencies to support day-care providers to improve the health and development of children in their care? | 1 - I am very competent  2 - I have some of the competencies required  3 - I don’t feel I have the required competencies | 3 | 1,2 |
| ***Maximum component raw score = 3; Percent score = (Raw score/3)*100%*** | | | | |
|  | | | | |
| **Maximum total CHV KAPs raw score = 2+2+5+3+8+4+4+3= 31**  **Total CHV KAPs percent score = Mean of component percent scores (sum of all component percent scores/8)** | | | | |
